# Supplementary material for: Concomitant control of mechanical properties and degradation in resorbable elastomer-like materials using stereochemistry and stoichiometry for soft tissue engineering
Source: Nat Commun. 2021 Jan 19;12:446. doi: 10.1038/s41467-020-20610-5 (PMC7815890; doi:10.1038/s41467-020-20610-5)
Supplement: Supplementary file 1 — Supplementary Information [file 41467_2020_20610_MOESM1_ESM.pdf]

Supplementary Information

Concomitant Control of Mechanical Properties and Degradation in Resorbable Elastomer-like Materials Using Stereochemistry and Stoichiometry for Soft Tissue Engineering

Mary Beth Wandel[1#], Craig A. Bell[2,3,4#], Jiayi Yu[1#], Maria C. Arno[5], Nathan Z. Dreger[1], Yen-Hao Hsu[1], Anaïs Pitto-Barry[2], Joshua C. Worch[5], Andrew P. Dove[*5], Matthew L. Becker[*1,6]

[1] Department of Polymer Science, The University of Akron, Akron, OH 44325

[2] Department of Chemistry, The University of Warwick, Coventry, CV4 7AL, UK

[3] Centre for Advanced Imaging, The University of Queensland, St Lucia, Queensland 4072, Australia

[4] Australian Institute for Bioengineering and Nanotechnology, The University of Queensland, St Lucia, Queensland 4072, Australia

[5] School of Chemistry, The University of Birmingham, Edgbaston, Birmingham, B15 2TT, UK

[6] Department of Chemistry, Mechanical Engineering and Materials Science, Orthopaedic Surgery, Duke University, Durham, NC 20899

*E-mail: matthew.l.becker@duke.edu; a.dove@bham.ac.uk

[#] These authors contributed equally to this work

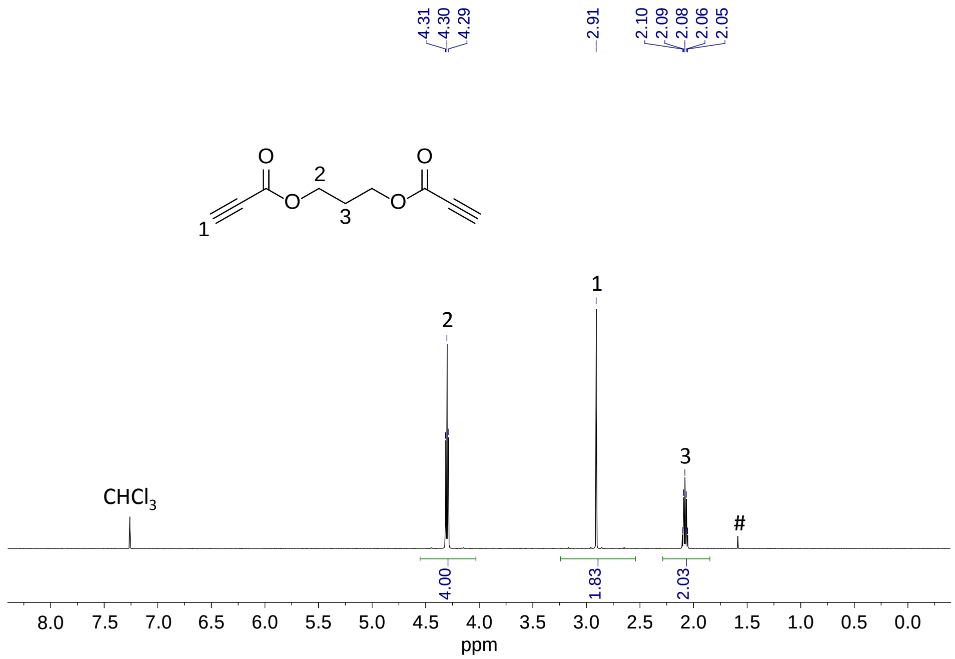

Figure 1 – ^{1}H NMR spectrum of **1** (500 MHz, $CDCl_3$). # – H_2O.

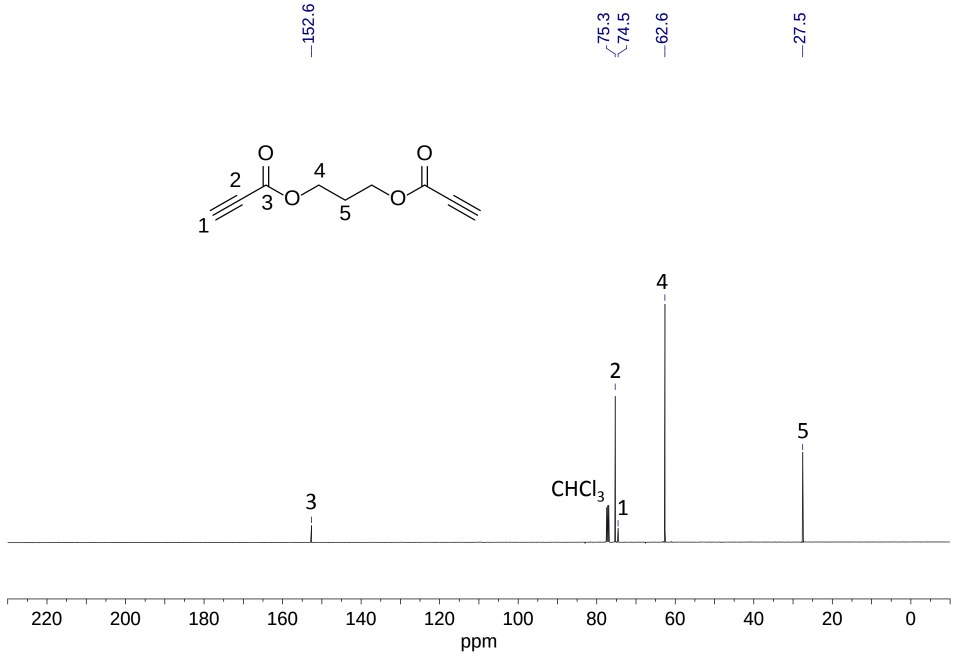

Figure 2 – ^{13}C NMR spectrum of **1** (125 MHz, $CDCl_3$).

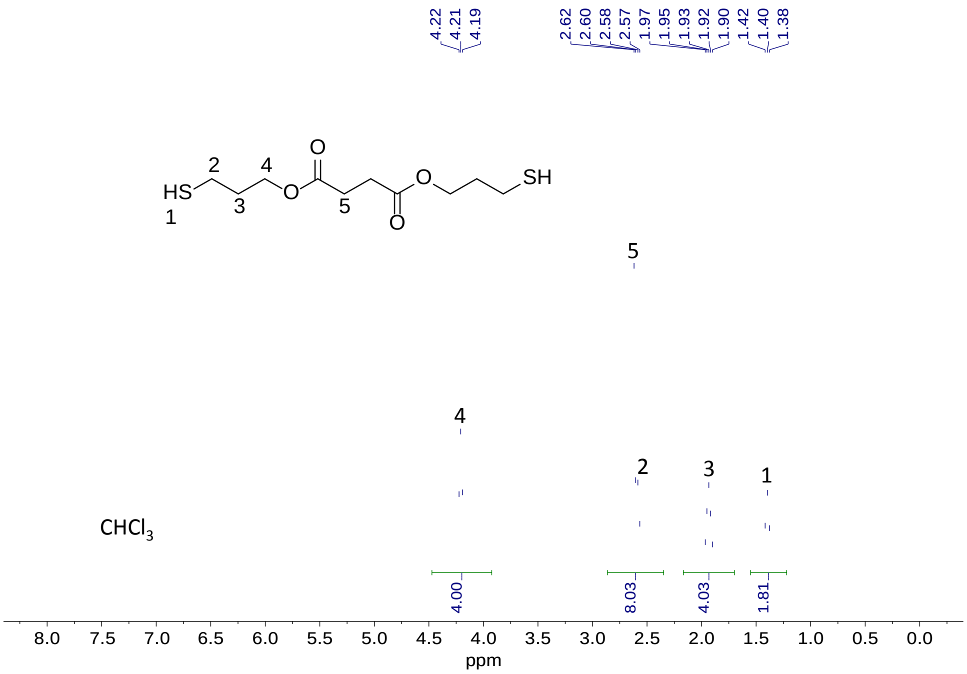

Figure 3 – ^{1}H NMR spectrum of **2** (400 MHz, $CDCl_3$).

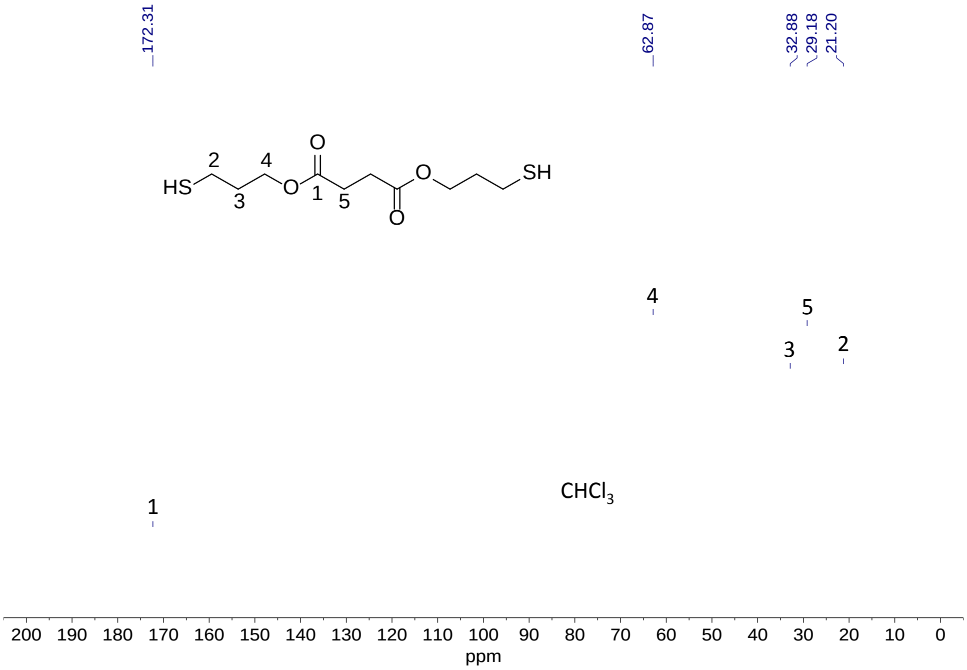

Figure 4 – ^{13}C NMR spectrum of **2** (100 MHz, $CDCl_3$).

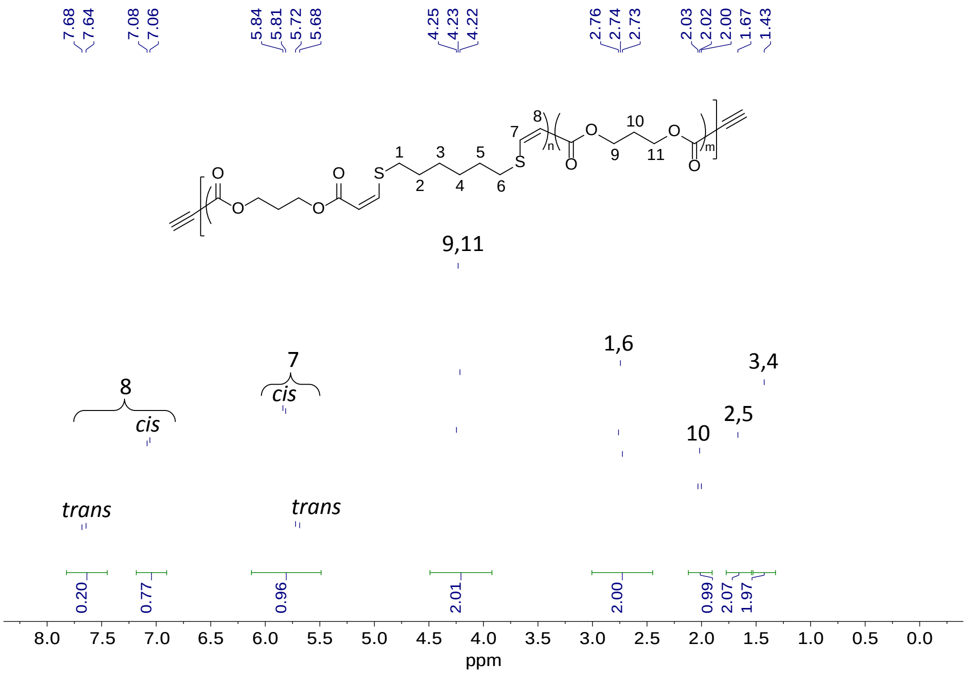

Figure 5 – ^{1}H NMR spectrum of C_3-C_6 thiol-yne step growth polymer with 0% incorporation of **2** (entry 1 in Table S1, 400 MHz, $CDCl_3$). % *cis* = 80%.

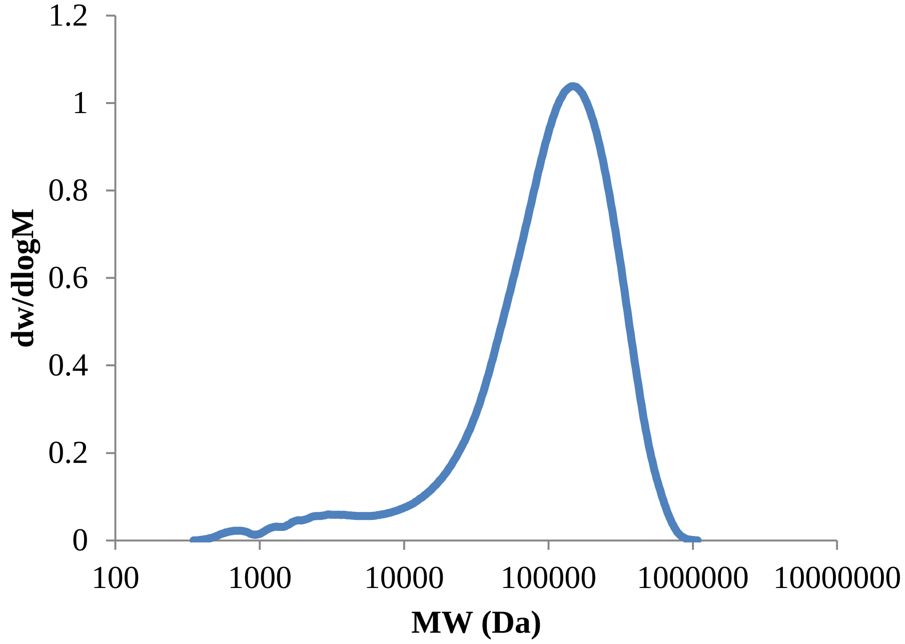

Figure 6 – SEC chromatogram of C_3-C_6 thiol-yne step growth polymer with 0% incorporation of **2** (entry 1 in Table S1); M_n = 26.4 kDa, M_w = 147.5, M_p = 150.4, $Đ_M$ = 5.60 (SEC $CHCl_3$ + 0.5% v/v Et_3N, based on PS standards).

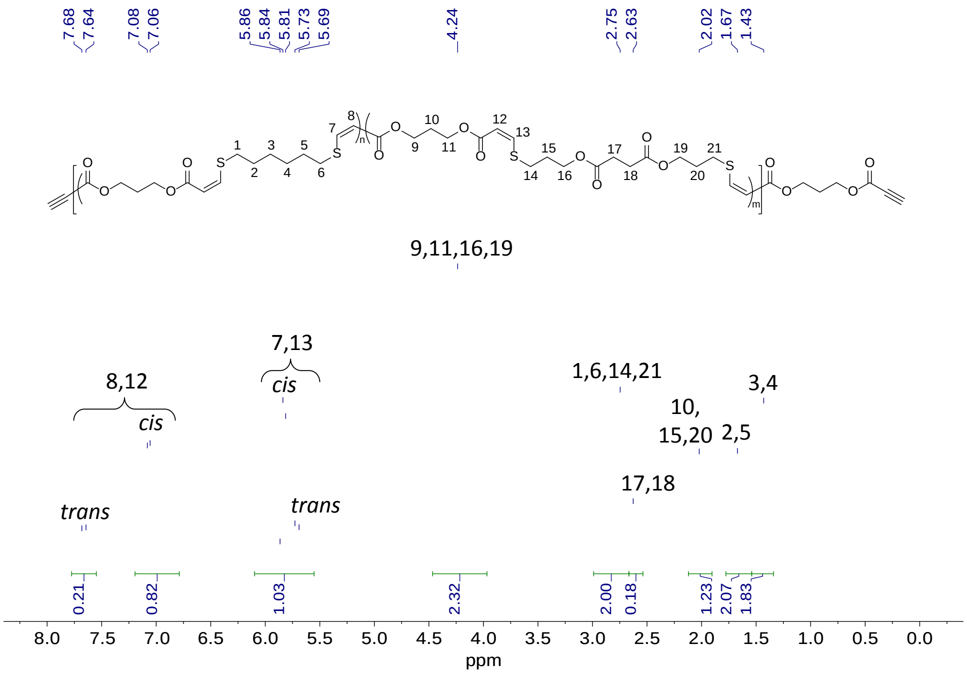

Figure 7 – ^{1}H NMR spectrum of C_3-C_6 thiol-yne step growth polymer with 9.0% incorporation of **2** (entry 2 in Table S1, 400 MHz, $CDCl_3$). % *cis* = 80%.

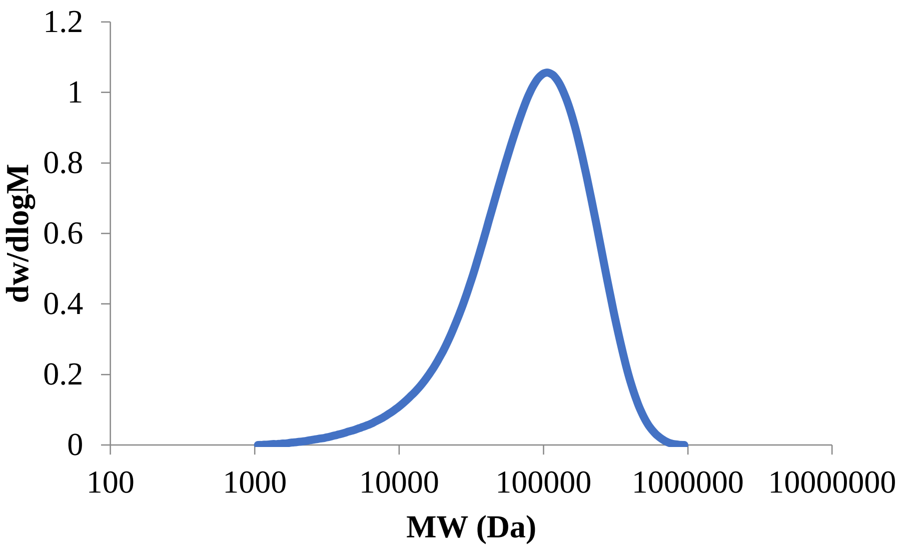

Figure 8 – SEC chromatogram of C_3-C_6 thiol-yne step growth polymer with 9.0% incorporation of **2** (entry 2 in Table S1); M_n = 29.7 kDa, M_w = 111.2, M_p = 106.9, $Đ_M$ = 3.74 (SEC $CHCl_3$ + 0.5% v/v Et_3N, based on PS standards).

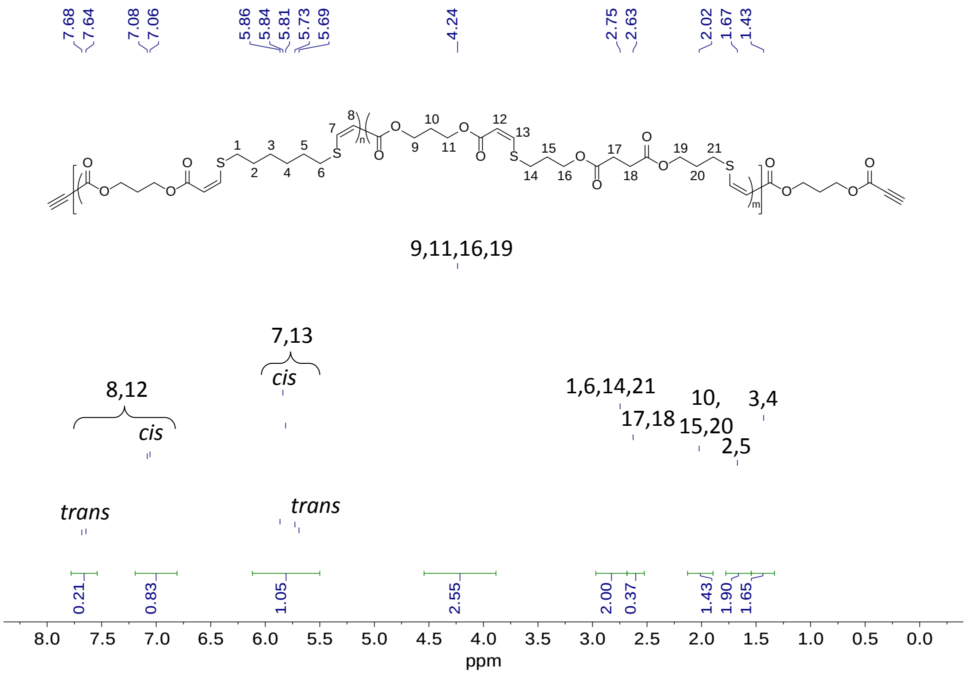

Figure 9 – ^{1}H NMR spectrum of C_3-C_6 thiol-yne step growth polymer with 18.7% incorporation of **2** (entry 3 in Table S1, 400 MHz, $CDCl_3$). % *cis* = 79%.

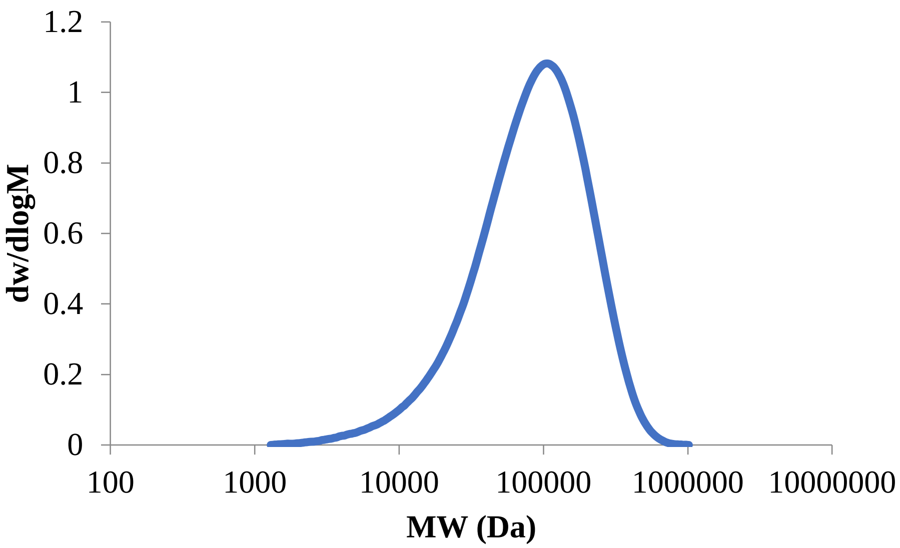

Figure 10 – SEC chromatogram of C_3-C_6 thiol-yne step growth polymer with 18.7% incorporation of **2** (entry 3 in Table S1); M_n = 35.2 kDa, M_w = 110.8, M_p = 106.9, $Đ_M$ = 3.15 (SEC $CHCl_3$ + 0.5% v/v Et_3N, based on PS standards).

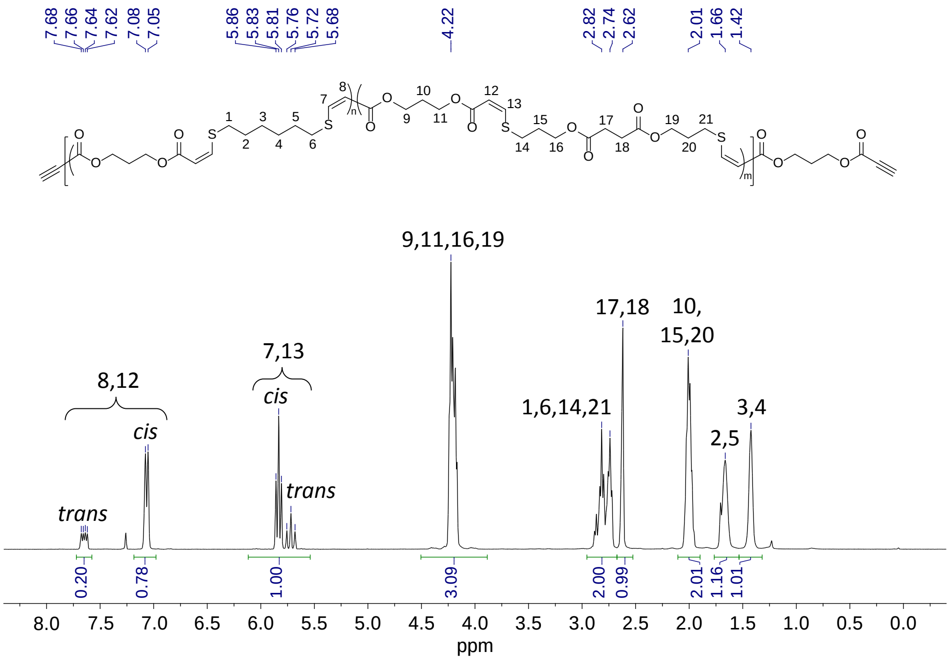

Figure 11 – ^{1}H NMR spectrum of C_3-C_6 thiol-yne step growth polymer with 49.4% incorporation of **2** (entry 4 in Table S1, 400 MHz, $CDCl_3$). % *cis* = 79%.

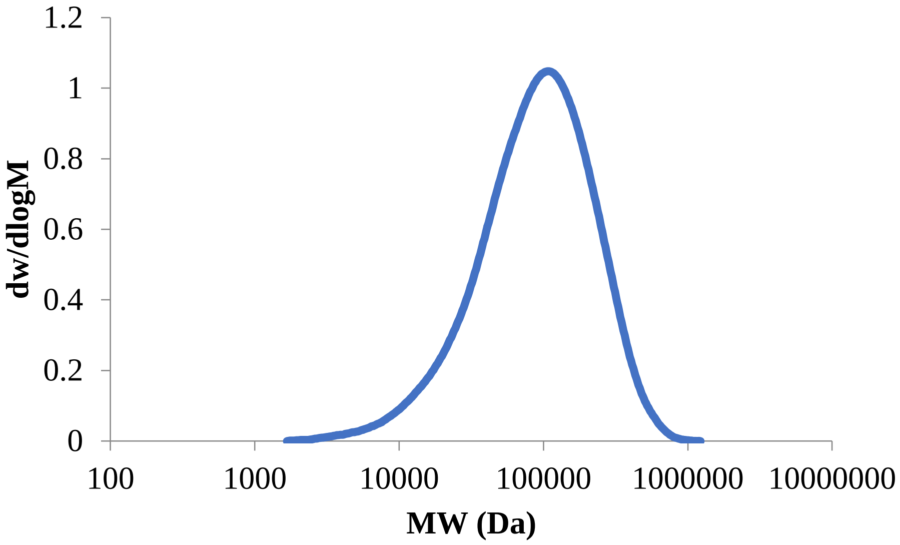

Figure 12 – SEC chromatogram of C_3-C_6 thiol-yne step growth polymer with 49.4% incorporation of **2** (entry 4 in Table S1); M_n = 52.5 kDa, M_w = 123.7, M_p = 109.7, $Đ_M$ = 2.36 (SEC $CHCl_3$ + 0.5% v/v Et_3N, based on PS standards).

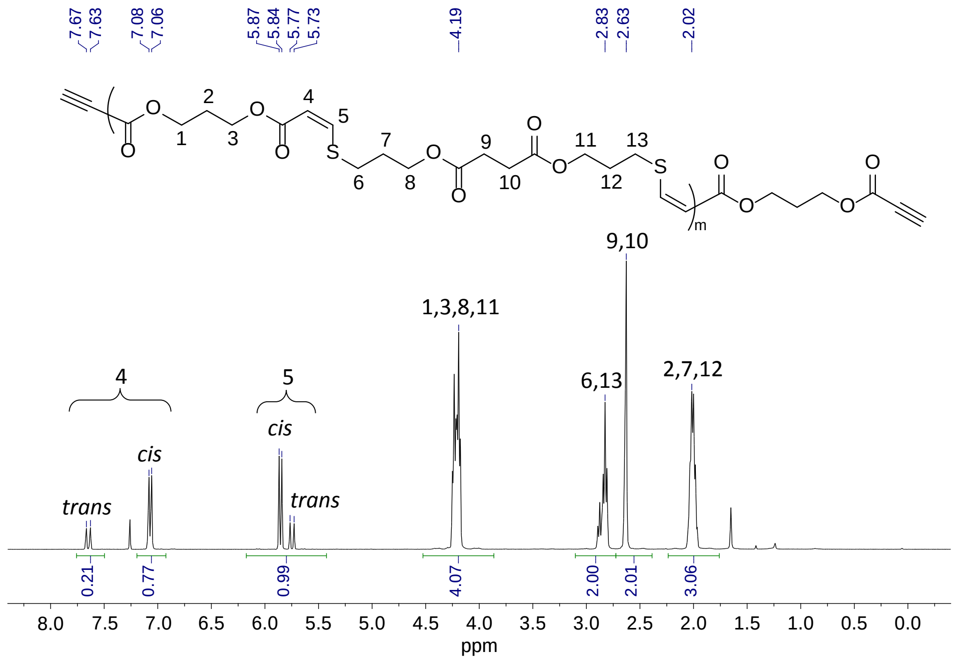

Figure 13 – ^{1}H NMR spectrum of C_3-C_6 thiol-yne step growth polymer with 100% incorporation of **2** (entry 5 in Table S1, 400 MHz, $CDCl_3$). % *cis* = 79%.

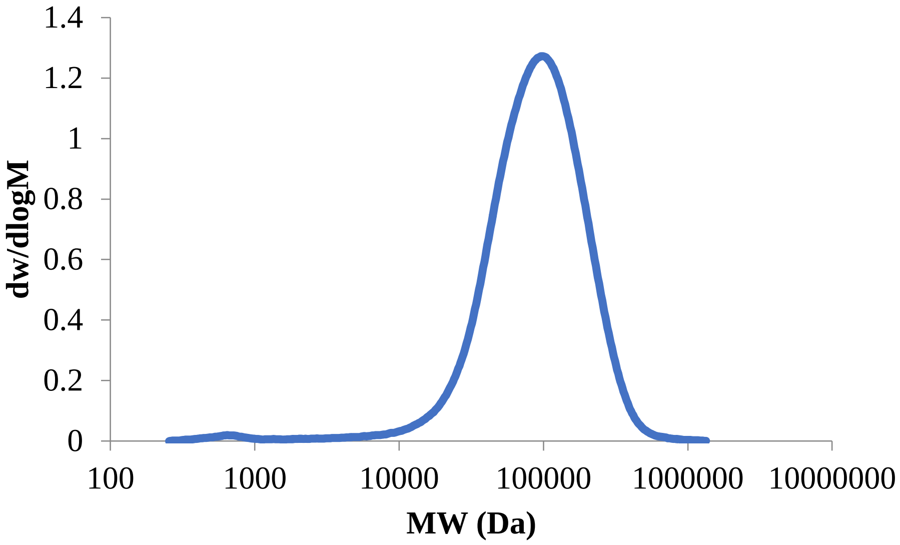

Figure 14 – SEC chromatogram of C_3-C_6 thiol-yne step growth polymer with 100% incorporation of **2** (entry 5 in Table S1); M_n = 35.9 kDa, M_w = 112.2, M_p = 97.6, $Đ_M$ = 3.12 (SEC $CHCl_3$ + 0.5% v/v Et_3N, based on PS standards).

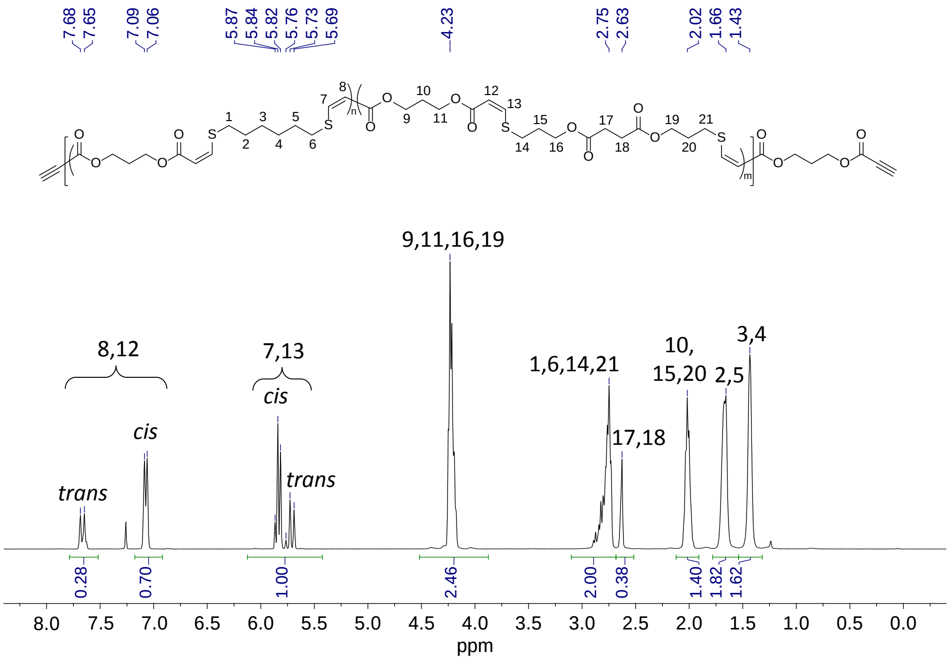

Figure 15 – ^{1}H NMR spectrum of C_3-C_6 thiol-yne step growth polymer with 19.0% incorporation of **2** (entry 6 in Table S1, 400 MHz, $CDCl_3$). % *cis* = 72%.

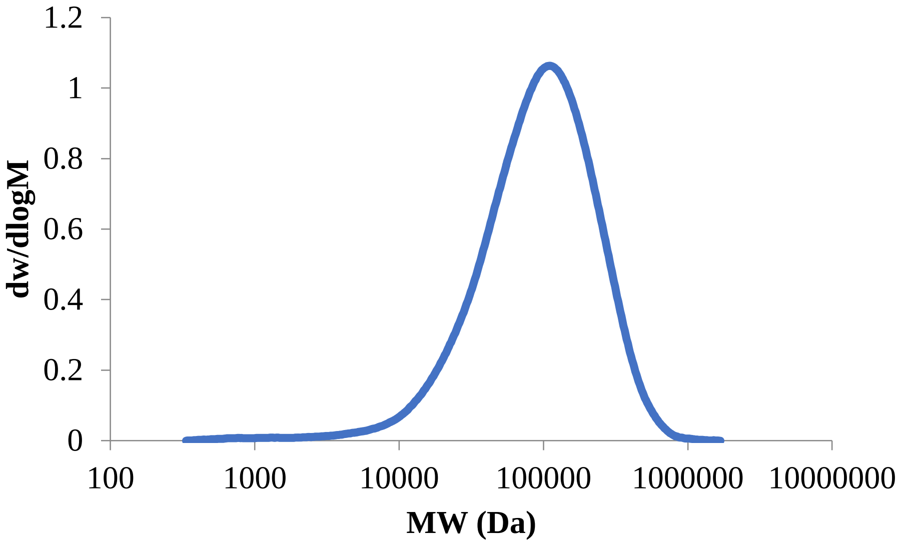

Figure 16 – SEC chromatogram of C_3-C_6 thiol-yne step growth polymer with 19.0% incorporation of **2** (entry 6 in Table S1); M_n = 43.0 kDa, M_w = 127.3, M_p = 109.7, $Đ_M$ = 2.96 (SEC $CHCl_3$ + 0.5% v/v Et_3N, based on PS standards).

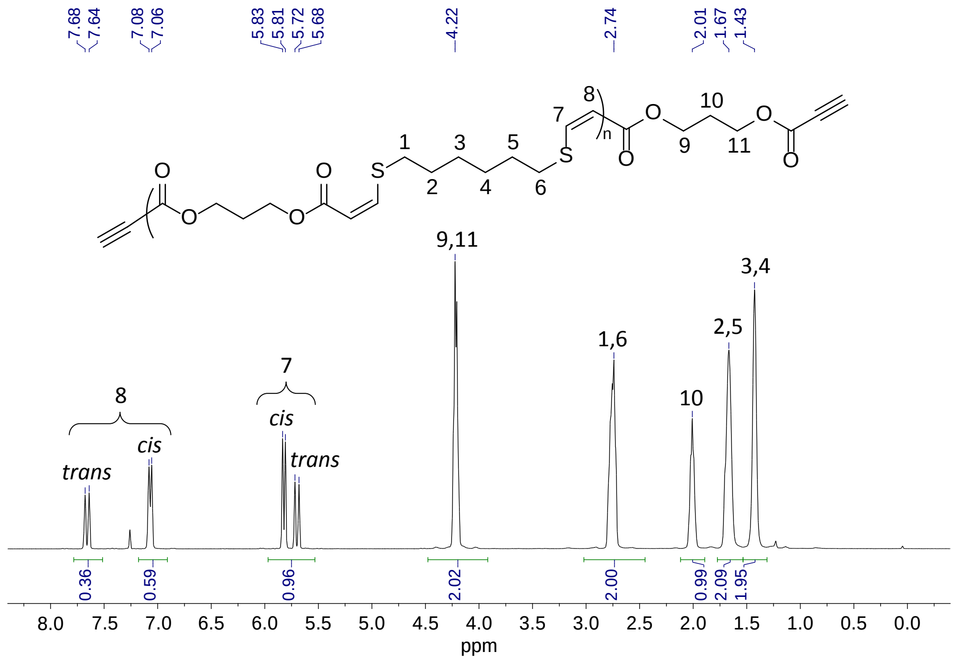

Figure 17 – ^{1}H NMR spectrum of C_3-C_6 thiol-yne step growth polymer with 0% incorporation of **2** (entry 7 in Table S1, 400 MHz, $CDCl_3$). % *cis* = 62%.

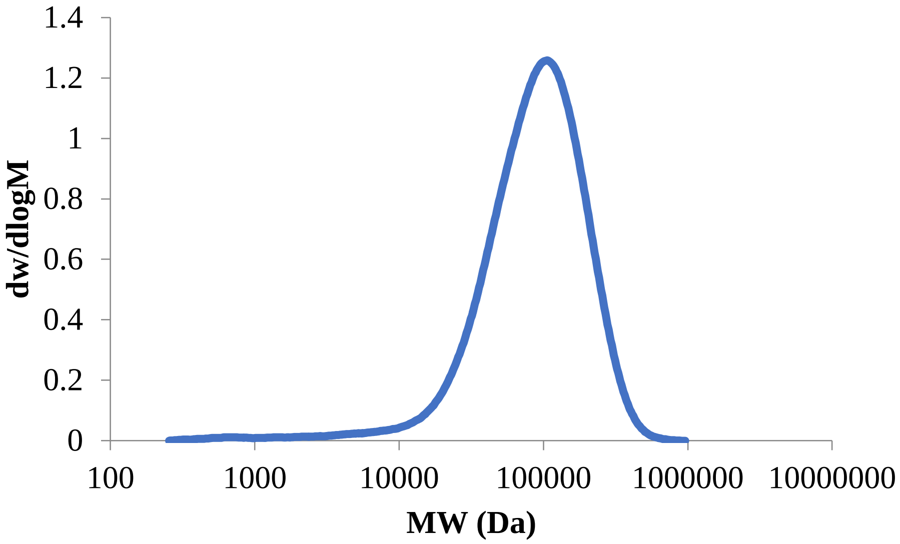

Figure 18 – SEC chromatogram of C_3-C_6 thiol-yne step growth polymer with 0% incorporation of **2** (entry 7 in Table S1); M_n = 37.0 kDa, M_w = 110.8, M_p = 106.1, $Đ_M$ = 3.00 (SEC $CHCl_3$ + 0.5% v/v Et_3N, based on PS standards).

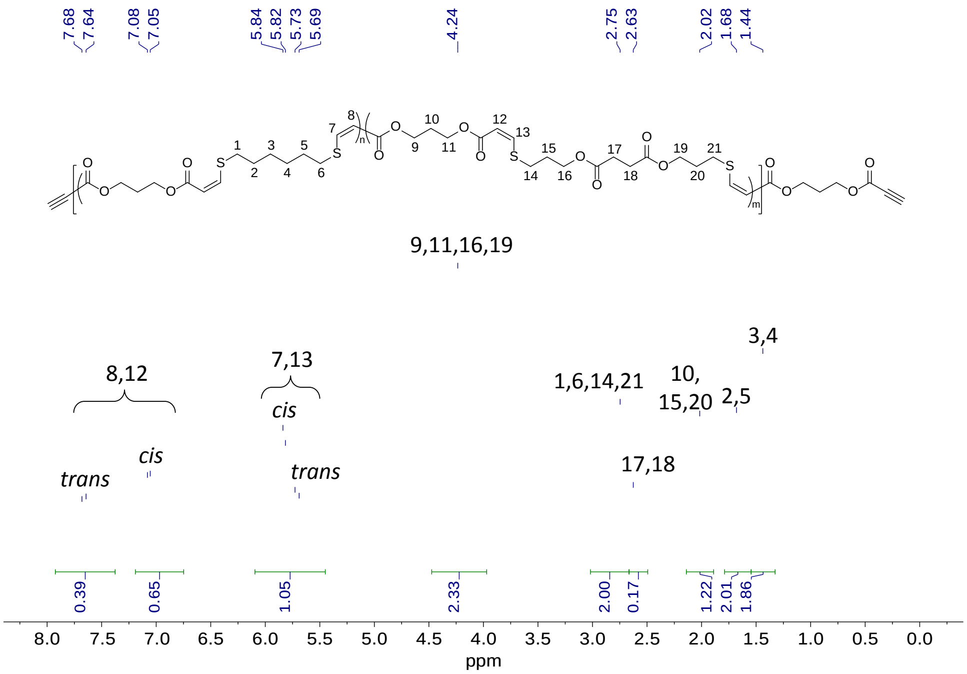

Figure 19 – ^{1}H NMR spectrum of C_3-C_6 thiol-yne step growth polymer with 8.7% incorporation of **2** (entry 8 in Table S1, 400 MHz, $CDCl_3$). % *cis* = 62%.

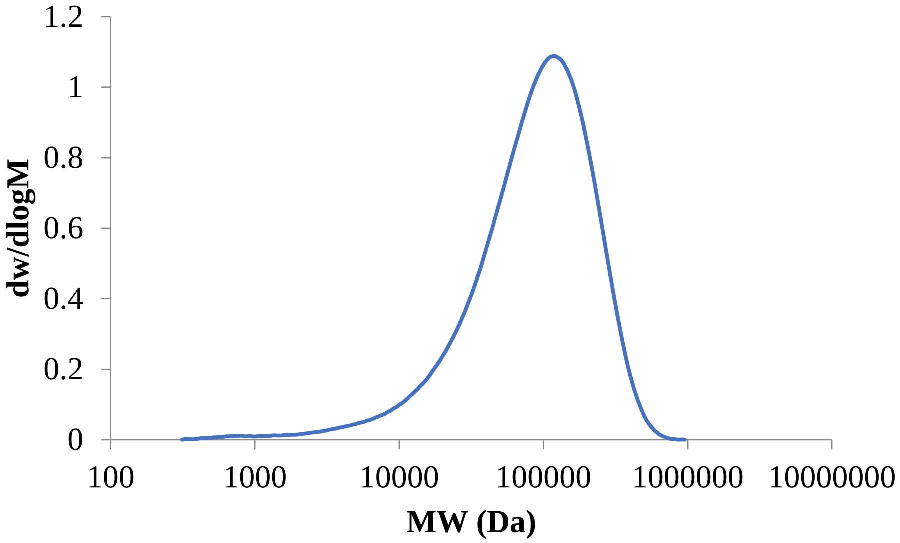

Figure 20 – SEC chromatogram of C_3-C_6 thiol-yne step growth polymer with 8.7% incorporation of **2** (entry 8 in Table S1); M_n = 34.2 kDa, M_w = 117.1, M_p = 119.6, $Đ_M$ = 3.42 (SEC $CHCl_3$ + 0.5% v/v Et_3N, based on PS standards).

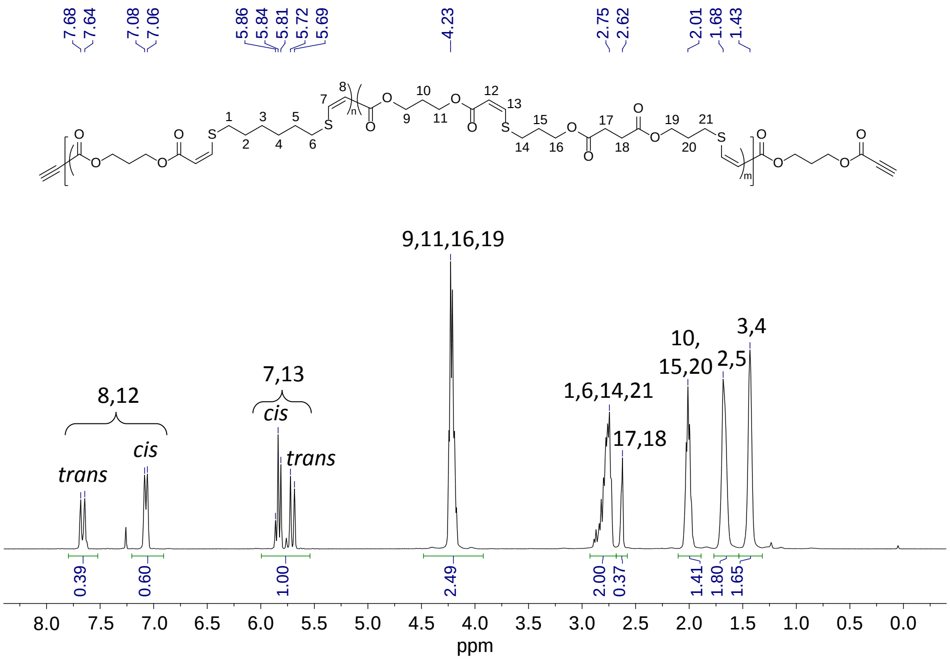

Figure 21 – ^{1}H NMR spectrum of C_3-C_6 thiol-yne step growth polymer with 18.3% incorporation of **2** (entry 9 in Table S1, 400 MHz, $CDCl_3$). % *cis* = 61%.

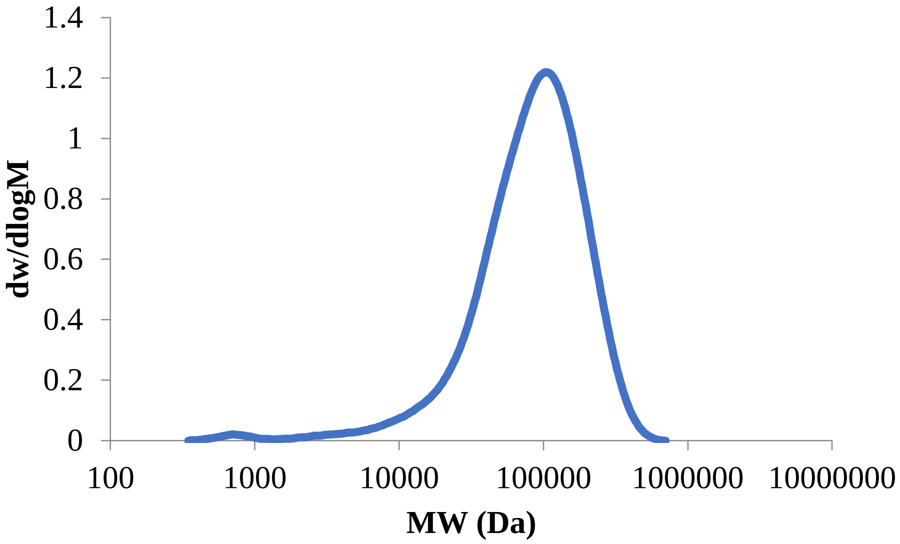

Figure 22 – SEC chromatogram of C_3-C_6 thiol-yne step growth polymer with 18.3% incorporation of **2** (entry 9 in Table S1); M_n = 35.3 kDa, M_w = 107.8, M_p = 104.4, $Đ_M$ = 3.05 (SEC $CHCl_3$ + 0.5% v/v Et_3N, based on PS standards).

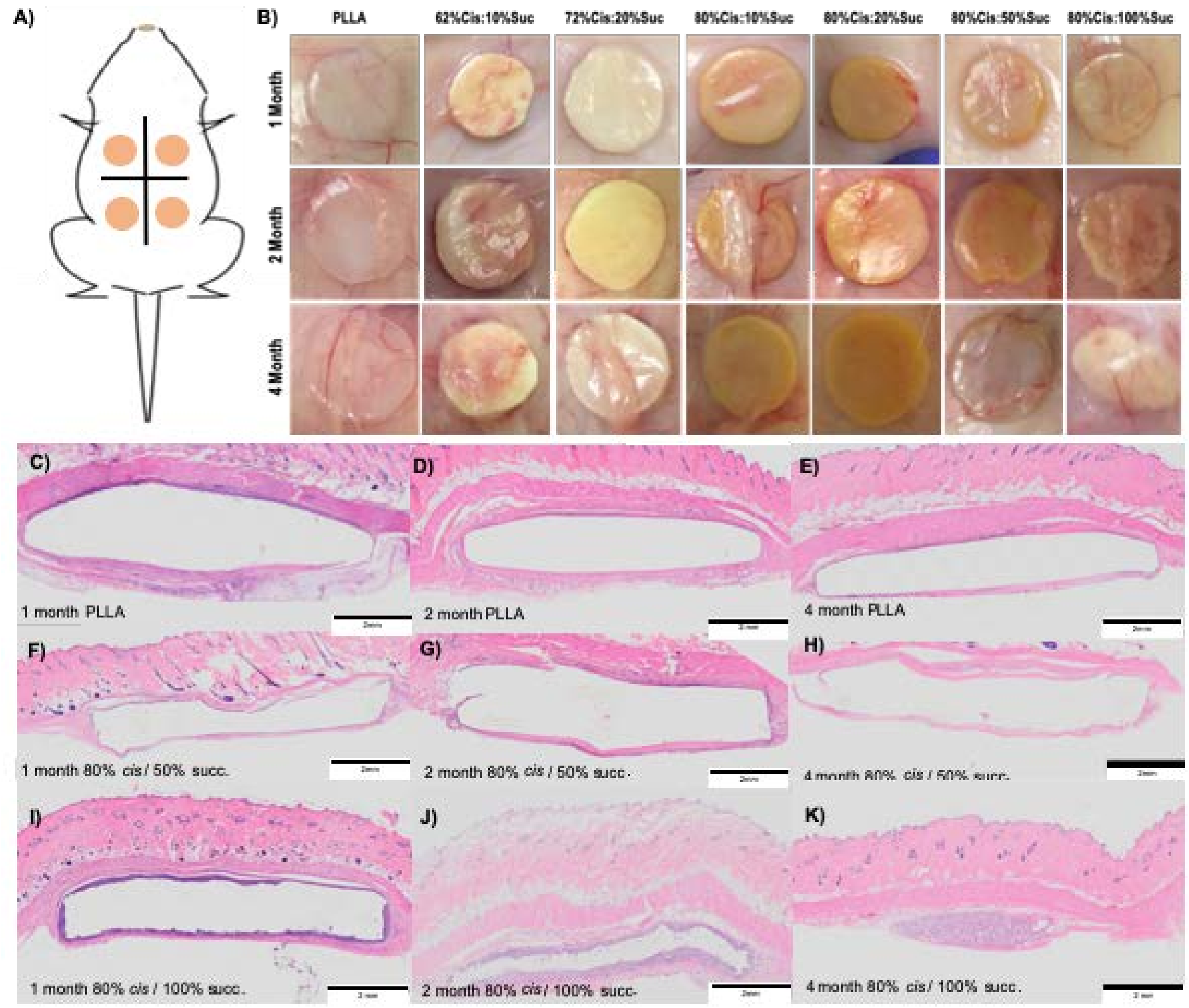

Figure 23. Subcutaneous *in vivo* degradation of PLLA, 80% *cis* / 50% succinate and 80% *cis* / 100% succinate over a 4-month timeframe. Surgical procedures with subcutaneous implantation involved a small incision, polymer disc insertion, and incision closure with Michel-clips. Four samples were implanted per animal (A). (B) Following extraction, the implants can be visualized in the host tissue. As seen, there are almost no macroscopic indications of an inflammatory response. Whole-mount cross-section image showing thick fibrous encapsulation surrounding PLLA after 4 months of incubation *in vivo* can be observed (C-E). Similar behavior to PLLA is observed for 80% cis / 50% succinate through 4-months implantation (F-H). Alternatively, the early stages of cellular infiltration are noticed in 80% *cis* / 100% succinate after only 1 month (I). After 2 months, noticeable shrinking/resorption of the elastomer was seen with continued cellular infiltration (J). Degradation after 4 months is nearly complete with cells and tissue fully encompassing the polymer area (K). Blood vessel sprouts and multinucleated giant cells are noticeable throughout the polymer space that has been resorbed. Inset scale bar = 2 mm.

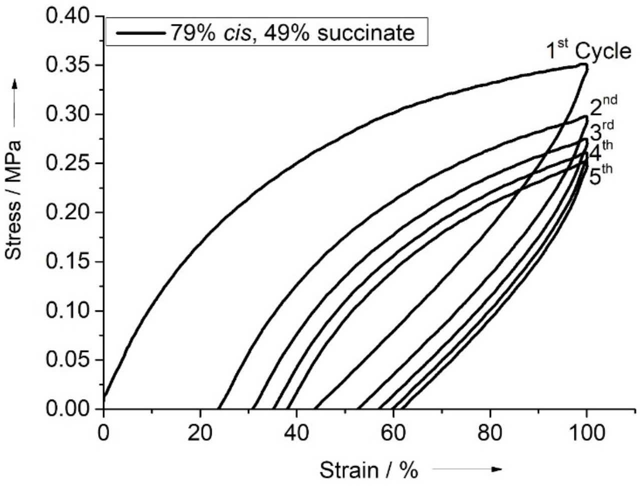

Figure 24. The hysteresis was performed by load-unloaded cyclic stress vs. strain curves stretching up to 100% with 5 cycles at 10 mm/min strain rate.

REFERENCES

1. B. M. Mandal, *Fundamentals of Polymerization*, World Scientific, 2013.
2. V. X. Truong and A. P. Dove, *Angew. Chem. Int. Ed.*, 2013, **52**, 4132 –4136.
